# Population Dynamics of Bank Voles Predicts Human Puumala Hantavirus Risk

**DOI:** 10.1007/s10393-019-01424-4

**Published:** 2019-07-15

**Authors:** Hussein Khalil, Frauke Ecke, Magnus Evander, Göran Bucht, Birger Hörnfeldt

**Affiliations:** 1 Department of Wildlife, Fish, and Environmental Studies, Swedish University of Agricultural Sciences, 901 83 Umeå, Sweden; 2 Department of Aquatic Sciences and Assessment, Swedish University of Agricultural Sciences, P.O. Box 7050, 750 07 Uppsala, Sweden; 3 Department of Clinical Microbiology, Virology, Umeå University, 901 85 Umeå, Sweden; 4 Swedish Defense Research Agency, CBRN Defence and Security, Umeå, Sweden

**Keywords:** Bank vole, Disease dynamics, Epidemiology, Hantavirus, Landscape, Nephropathia epidemica, Puumala virus, Sweden

## Abstract

Predicting risk of zoonotic diseases, i.e., diseases shared by humans and animals, is often complicated by the population ecology of wildlife host(s). We here demonstrate how ecological knowledge of a disease system can be used for early prediction of human risk using Puumala hantavirus (PUUV) in bank voles (*Myodes glareolus*), which causes *Nephropathia epidemica* (NE) in humans, as a model system. Bank vole populations at northern latitudes exhibit multiannual fluctuations in density and spatial distribution, a phenomenon that has been studied extensively. Nevertheless, existing studies predict NE incidence only a few months before an outbreak. We used a time series on cyclic bank vole population density (1972–2013), their PUUV infection rates (1979–1986; 2003–2013), and NE incidence in Sweden (1990–2013). Depending on the relationship between vole density and infection prevalence (proportion of infected animals), either overall density of bank voles or the density of infected bank voles may be used to predict seasonal NE incidence. The density and spatial distribution of voles at density minima of a population cycle contribute to the early warning of NE risk later at its cyclic peak. When bank voles remain relatively widespread in the landscape during cyclic minima, PUUV can spread from a high baseline during a cycle, culminating in high prevalence in bank voles and potentially high NE risk during peak densities.

## Introduction

The emergence and re-emergence of virulent human pathogens over the past two decades (Kilpatrick and Randolph 2012) increased alertness to the global burden of infectious diseases originating in wildlife. Changes in scale and distribution of such diseases were typified by recent high-profile outbreaks of Ebola virus in West Africa (Spengler et al. 2016) and the introduction of West Nile virus in North America (Jones et al. 2008).

For many vector-borne and zoonotic diseases, multiple species with different life histories and population dynamics are involved in the sequence of transmission events that lead to human infections. Hosts and vectors commonly show discernible seasonal (Altizer et al. 2006) and annual or multiannual variation (Ostfeld et al. 2006) in abundance and infection rates. Often, changes in host abundance are accompanied by behavioral changes driven by factors inherent to host populations, e.g., life history and demographic traits (Olsson et al. 2003a; Fichet-Calvet et al. 2014). To mitigate zoonotic risk, a system-level understanding of the ecology of the disease system—constituted by host, vector, and pathogen—is pertinent (Mills and Childs 1998).

Rodents are important hosts of zoonotic diseases (Han et al. 2015). Globally, rodent-borne zoonotic pathogens cause a plethora of diseases, including hemorrhagic fevers caused by hantaviruses and arenaviruses. Many rodent populations exhibit great spatial and temporal fluctuations in density (Krebs and Myers 1974; Davis et al. 2005). These fluctuations include annual (Singleton et al. 2001) and multiannual population cycles, the latter typical of northern latitudes (Krebs 1996). Over the course of a multiannual cycle, small mammal density can vary by several orders of magnitude (Krebs 1978; Hörnfeldt 1978; Hansson and Henttonen 1985; Hörnfeldt 1994). Fluctuations in abundance are often accompanied by changes in spatial distribution (e.g., Khalil et al. 2014b; Hörnfeldt et al. 2006; Carver et al. 2015), leading to pronounced changes in zoonotic risk over local spatial and short temporal scales (Ostfeld et al. 2005).

In Europe, rodents are responsible for thousands of annual cases of hemorrhagic fever with renal syndrome (HFRS; Vaheri et al. 2013), caused by two pathogenic species of the genus *Hantavirus*. In central and eastern Europe, yellow-necked and wood mice (*Apodemus* spp.) are carriers of Dobrava hantavirus (Nemirov et al. 1999), which causes most cases of hemorrhagic fever in the region. In contrast, bank voles (*Myodes glareolus*) carry Puumala hantavirus (PUUV) (Brummer-Korvenkontio et al. 1980), which causes a mild form of HFRS in humans known as *Nephropathia epidemica* (NE). PUUV is responsible for NE in Russia, Central and Western Europe, Finland, and northern Scandinavia (Olsson et al. 2010). Humans get infected upon the inhalation of aerosolized viral particles (Vapalahti et al. 2010; Vaheri et al. 2013).

The transmission of PUUV in bank voles is horizontal via direct contact or through the environment (Hardestam et al. 2008). Infection and shedding of viral particles in bank voles are chronic (Voutilainen et al. 2015). Viral RNA is secreted and excreted in saliva, urine, and feces. Viral shedding reaches its peak within a month after infection (Hardestam et al. 2008), yet remains relatively high afterward (Voutilainen et al. 2015). Infected females transfer maternal antibodies to their offspring, providing protection for up to 3 months (Kallio et al. 2010). The relationship between host and infected host densities is described by infection prevalence, i.e., proportion of infected animals, and depends on how transmission rates respond to changes in host density and demography (Begon et al. 2002; Davis et al. 2005). At elevated densities, contact rates or duration of contacts between infected and susceptible individuals may increase, resulting in a higher rate of transmission (reviewed in Khalil et al. 2014a for hantaviruses). However, transmission may be frequency dependent (McCallum et al. 2001), implying that contact rate among susceptible and infectious individuals remains constant regardless of changes in density. In such a scenario, the density of infected animals may increase with overall host density, but prevalence does not. Realistically, transmission does not necessarily conform exactly to density or frequency dependence. It can take alternative forms or be appropriately described through network models of contact (e.g., Olinky and Stone 2004). Nevertheless, if prevalence in bank voles increases with density, an increase in density would result in an exponential increase in the number of infected animals (Davis et al. 2005).

The number of annual cases of NE is closely linked to the abundance of bank voles in Finland (Kallio et al. 2009), Sweden (Niklasson et al. 1995; Olsson et al. 2009), and Central and Western Europe (Tersago et al. 2010; Reil et al. 2015). In Fennoscandia, most cases occur during winter, when infected bank voles infest human dwellings (Olsson et al. 2003a). In temperate Europe, NE risk can be predicted 2 years in advance based on weather conditions that promote high seed production from broad-leaved trees such as oak and beech. This “masting” phenomenon leads to subsequent bank vole population outbreaks (Tersago et al. 2009). At northern latitudes, small mammal cycles have intrigued ecologists for many decades, and a large body of literature is dedicated to understanding and explaining them (Krebs and Myers 1974; Hörnfeldt 1978, 1994, 2004; Hansson and Henttonen 1985; Hörnfeldt et al. 2005; Cornulier et al. 2013; Korpela et al. 2014; Magnusson et al. 2015a; Pöysä et al. 2016). However, despite extensive knowledge on multiannual bank vole cycles, existing studies predict NE incidence only a few months before an outbreak (Kallio et al. 2009; Olsson et al. 2009; Khalil et al. 2014b).

Here, we use a long time series on cyclic bank vole populations, their PUUV infection rates, and NE incidence in northern Sweden to infer seasonal and multiannual transmission dynamics within bank vole populations. We demonstrate how ecological and epidemiological aspects of a disease system can be combined for early warning of NE risk. We further discuss based on the seasonal relationship between bank vole density and their infection prevalence, whether seasonal infection risk in humans is better predicted by overall bank vole density or by the density of infected animals.

## Methods

### Bank Vole Data

**Figure 1:**
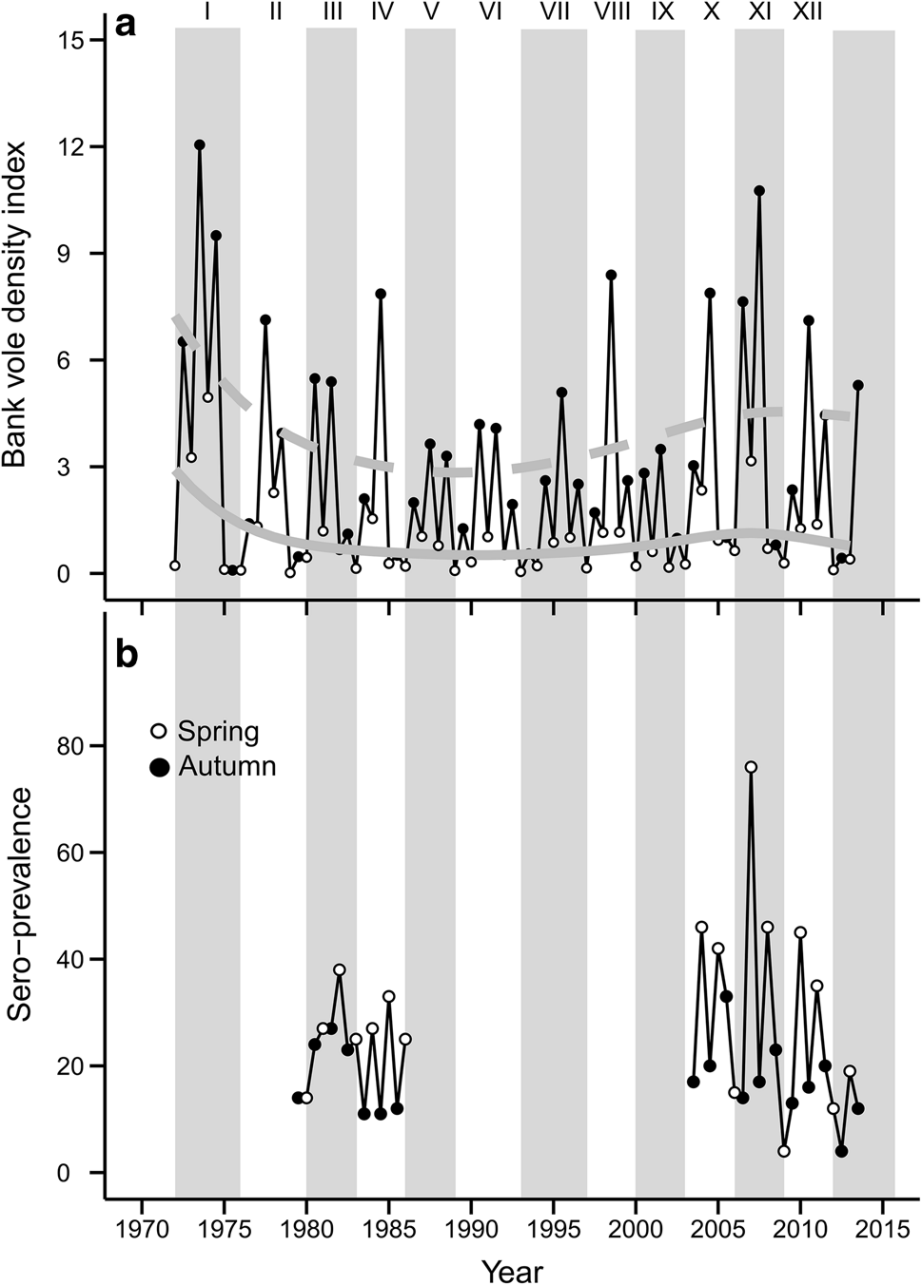
**a** Bank vole density index (number of trapped individuals per 100 trap nights) in 1972–2013 and **b** Puumala virus (PUUV) seroprevalence in spring (open circles) and autumn (filled circles). Data on PUUV seroprevalence are from autumn 1979 to spring 1986 and autumn 2003 to autumn 2013. No infection data were available in 1971–1978 and 1987–2002. Alternate shadings indicate different cycles, numbered I–XII. The gray lines in **a** represent the fitted GAM models (solid for spring and dashed for autumn).

Bank vole density data were available in spring and autumn 1972–2013 through the ongoing Swedish National Environmental Monitoring Program for small rodents near the city of Umeå in northern Sweden (64° N, 20° E) (Hörnfeldt 1994). Snap trapping of small mammals takes place twice a year within a 100 × 100 km area in 58 permanent and systematically placed 1-ha plots at least 2.5 km apart. There are ten trapping stations per 1-ha plot, each station with five traps within a circle of a 1 m radius. Spring trapping and autumn trapping occur in late May and late September, respectively, for three consecutive nights. The total effort per plot in a session is 150 trap nights (see Hörnfeldt 1978, 1994, 2004 for further details). For spring and autumn seasons, we calculated an index of bank vole density as the overall number of bank voles trapped per 100 trap nights, hereafter referred to as bank vole density. The bank vole time series spanned 12 complete cycles in population density (numbered I–XII; see Magnusson et al. 2015a) and exhibited marked differences in amplitude and peak numbers during the 43-year period (Fig. 1a) (Hörnfeldt 1994, 2004). A vole cycle generally comprises 3–4 years and is characterized by the following phases in density: increase, peak, decrease, and low (Hörnfeldt 1994).

### Ethics Statement

Trapping of animals was approved by the Swedish Environmental Protection Agency (latest permission: NV-01124-15) and the Animal Ethics Committee in Umeå (latest permissions: Dnr A 61-11 and A121-11), and all applicable institutional and national guidelines for the use of animals were followed.

### PUUV Infection Data in Bank Voles

Data on PUUV infection in bank voles were available in autumn 1979–1986 and 2003–2013 and spring 1980–1986 and 2004–2013 (see further Magnusson et al. 2015b; Khalil et al. 2016). We analyzed lung samples from bank voles by enzyme-linked immunosorbent assay (ELISA) to detect anti-PUUV IgG antibodies and identify seropositive individuals (Lindkvist et al. 2008; Khalil et al. 2016). The detection threshold in the PUUV IgG ELISA was determined by analyzing 32 sera, previously confirmed PUUV negative by immunofluorescence assay. The assay threshold was determined by counting the mean optical density value of these PUUV-negative samples + 3 standard deviations. Seropositive bank voles weighing < 14.4 g may carry maternal antibodies (Kallio et al. 2006; Voutilainen et al. 2012) and were excluded from further analyses as their status may not reflect genuine infection (*n* = 348 in 1979–1986; *n* = 902 in 2003–2013). PUUV infection in bank voles is chronic and infected individuals shed the virus throughout their life (Voutilainen et al. 2015), so we considered seropositive individuals infected.

Subsequent analyses of available PUUV infection data were based on 2064 and 4294 bank voles in 1979–1986 and 2003–2013, respectively. We calculated prevalence for spring and autumn seasons (see above) for which infection data were available as the percentage of infected animals trapped in that season (number of infected bank voles/overall number of bank voles) × 100. Data on bank vole abundance are available through the national environmental monitoring Web site [In Swedish]: https://www.slu.se/institutioner/vilt-fisk-miljo/miljoanalys/miljoovervakning-av-smagnagare/.

### NE Incidence Data

NE has been a notifiable disease in Sweden since 1989, i.e., NE cases are diagnosed by accredited clinical microbiology laboratories and must be reported to the Public Health Agency of Sweden. Human incidence data for the four northernmost counties, where more than 90% of NE cases occur (Olsson et al. 2003a), were available from the Public Health Agency of Sweden Web site (https://www.folkhalsomyndigheten.se/ [*In Swedish]*). The data were available at county level, and NE incidence (no. cases per 100,000 inhabitants) was divided into two periods: spring–summer period (April–September, hereon referred to as summer) and autumn–winter period (October–March, hereon referred to as winter and the year for that season refers to the year in October, when the season started). The time periods for incidence were chosen such that bank vole density and infected bank vole density in spring and autumn would be used to predict NE incidence over two six-month periods (summer and winter). Since spring trapping and autumn trapping of bank voles are separated by 4 months only (late May to late September), we included April and May in summer incidence. To investigate the potential bias in predictions arising from our classification of summer and winter periods, we compared the results from the original classification with those using a different classification, with summer incidence defined as May–October or June–November and winter as September–February or November–April. The results were qualitatively the same (SI Table 1). Hence, we persisted with the original periods: summer being April–September and winter being October–March. In subsequent analyses, we used NE incidence data in 1990–2013, as preliminary analysis suggested that the first year of reporting, 1989, was a negative outlier with NE incidence being much lower than expected given bank vole density in the same year (Khalil et al. 2014b).

**Table 1:** Sensitivity Analysis for *Nephropathia epidemica* Classification of Summer and Winter Seasons.

NE summer	Bank vole density (%)	Infected bank vole density (%)
*Spring*		
April–September	88	87
May–October	92	88
June–November	92	91
*Autumn*		
October–March	77	45
September–February	80	49
November–April	70	38

We evaluated how the proportion of explained variation (GLM, pseudo *R*^2^) in seasonal *Nephropathia epidemica* incidence (NE) changes with the classification of summer and winter time periods.

### Statistical Analyses

#### Long-Term Trends in Bank Vole Density

All statistical analyses were performed in R statistical software (R Core Team 2016) using base R except when otherwise stated.

To describe the long-term changes in bank vole populations in 1972–2013, we fitted generalized additive models (GAM) with year as the explanatory variable to spring and autumn vole densities using “mgcv” package (Wood 2011). We used GAMs (Zuur et al. 2009), adjusted for over-dispersion and non-integer values through a quasi-Poisson error distribution. Because the observations in the time series were not independent, we used an autocorrelation function, with an order (lag) defined by parameter *p*. We included lags up to three years (*p *=3), the typical length of a bank vole population cycle (Hörnfeldt 1994), to account for previous population density in spring and autumn (for spring and autumn models, respectively). We compared the models with different time lags (Hefley et al. 2017) using an adjusted form of the Akaike Information Criterion (AIC), qAIC, because we used quasi-Poisson models (Burnham and Anderson 2002; Ver Hoef and Boveng 2007). If two or more models differed by two or less qAIC units, we chose the simplest. The fitted GAM model for each season was:

$$ \ln \left( {\mu_{i,j} } \right) = f \left( {\text{Year}} \right)_{j} + \mathop \sum \limits_{p = 1}^{3} \emptyset_{i,j} \times D_{i,j - p} $$

where $\mu_{i,j}$ is the expected value of the bank vole density ($D$) during the $i$th season of the $j$th year, *f* (Year)_j_ is the effect for the $j$th year from a smoothing function over the years of the study, and $\emptyset_{i,j}$ is the effect of bank vole density from the $p$th previous season $i$ (lag effect).

We followed the same procedure for the subsequent GAM models. We also checked for temporal confounding between the basis vector for the smoothed parameter: year, and the lags we included in the model. Concurvity is the nonparametric analog of multicollinearity and may lead to underestimation of the variance model parameters and thus type 1 errors (Hefley et al. 2017). The models for NE incidence and bank vole density all had concurvity scores < 0.1 and thus did not show temporal confounding. For all models, we checked for autocorrelation and nonlinear patterns in the residuals.

#### Bank Vole Density and Infection Prevalence

PUUV prevalence in bank voles was a proportion, and hence, for both spring and autumn seasons, we fitted a generalized linear model with binomial error distribution and a logit link function using PUUV prevalence in bank voles as the dependent variable. Vole density during the same season and that during the previous season were the explanatory variables, to test for both direct and delayed density dependence (Niklasson et al. 1995). The model was the following:

$$ {\text{logit}} \left( P \right) = \beta_{0} \times D_{i} + \emptyset_{i} \times D_{i - 1} $$

where $$ P $$ is the probability of a bank vole being PUUV positive, *β*_0_ is the effect of $$ \left( D \right) $$ bank vole density in season $$ i $$, and $$ \emptyset_{i} $$ is the effect of bank vole density $$ \left( D \right) $$ in the previous season ($$ i - 1). $$

We also calculated PUUV infection prevalence in bank voles in spring and autumn between two distinct time periods with different bank vole densities (cf. Khalil et al. 2016), spanning a total of five population cycles. During the earlier period: 1979–1986 (cycles III and IV, *n*_bank voles_ = 2412), bank vole densities were lower than during the latter period (see above): 2003–2013 (cycles X–XII, *n*_bank voles_ = 5196). We also used *F*-ratio tests to compare the variance in prevalence in 1979–1986 with variance in prevalence in 2003–2013.

#### Explaining Seasonal NE Incidence in Humans

We investigated long-term changes in NE incidence in summer and winter in 1990–2013. We fitted GAM models with year as explanatory variable using a quasi-Poisson error structure and log link function and included an autocorrelation process to the residuals, with an order (lag) of up to 3 years. The lag represented NE incidence in the same season (summer or winter) in the previous 3 years:

$$ \ln \left( {\mu_{i,j} } \right) = f \left( {\text{Year}} \right)_{j} + \mathop \sum \limits_{p = 1}^{3} \emptyset_{i,j} \times I_{i,j - p} $$

where $\mu_{i,j}$ is the expected value of NE incidence ($I$) during the $i$th season of the $j$th year, *f* (Year)_j_ is the effect for the $j$th year from a smoothing function over the years of the study, and $\emptyset_{i,j}$ is the effect of NE incidence from the $p$th previous season $i$ (lag effect).

To evaluate whether overall host density or density of infected animals better predicts seasonal NE incidence, we compared using qAIC two models that used either overall density of bank voles or density of infected voles during the period 2003–2013. We studied NE incidence models for summer and winter separately, resulting in a total of four generalized linear models (GLM) with a quasi-Poisson error distribution and log link function. The models explaining NE incidence in each season were:

$$ \ln \left( {\mu_{i} } \right) = \beta_{0} \times D_{i} $$

where $\mu_{i}$ is the expected value of NE incidence in season $i$ and $\beta_{0}$ is the effect of *vole density* ($D$) in season $i$and

$$ \ln (\mu_{i} ) = \beta_{0} \times {\text{Dpos}}_{i} $$

where $\mu_{i}$ is the expected value of NE incidence in season $i$ and$\beta_{0}$ is the effect of *density of infected bank voles* (${\text{Dpos}}$) in season $i$.

#### Early Forecast of NE Risk

In the first spring of the vole cycle, densities are normally at a 3–4-year minimum, and a high reproductive output during the ensuing summer signals the beginning of a new cycle (Hörnfeldt 1994). In rare exceptions, this increase phase extends into a second year, as in 1980 when the initial increase in 1979 started from a very low density and yielded little numerical increase in absolute numbers. The population thus remained at very low density in autumn (Fig. 1a). We used bank vole density in spring of the increase phase (sensu Hörnfeldt 1994)—from the year when the population also attained a density of > 1 bank vole per 100 trap nights in autumn (in 1980, 1983, 2003, 2006, and 2009)—to predict the maximum density of infected voles reached during that cycle (five cycles: cycles: III–IV in 1979–1986 and X–XII in 2003–2013). We fitted a linear regression model and evaluated its predictive performance using predictive residual sum of squares (PRESS), despite the small sample size (*n* = 5). PRESS value is calculated by removing one observation from the data, fitting to the model to the remaining observations, and then using the regression function to predict the excluded value. The procedure then is repeated for all observations (*n* = 5), and subsequently a predictive *R*^2^ is calculated based on the PRESS values (Frost 2013). The predictive *R*^2^ indicates how good the model is in predicting left-out data points. The linear regression model was:

$$ \pi_{c} = \beta_{0} \times D_{c} $$

where $\pi_{c}$ is the expected value of maximum bank vole density in a vole population cycle ($c$) and $\beta_{0}$ is the effect of bank vole density ($D$) at the beginning of that cycle. We also correlated the proportion of the 58 1-ha plots occupied in spring of the increase phase as defined above with density of infected bank voles in spring of the following year, a peak year.

## Results

Bank vole density decreased during the 1980s and 1990s and then increased during the 2000s in both spring and autumn (Fig. 1a). Based on qAIC comparison (SI Table 2), the best model for each season included a temporal autocorrelation function with a two-year lag (GAM: *F*_39_ = 16.98; *p *< 0.001 for spring, *F*_39_= 7.645; *p* < 0.001 for autumn).

**Table 2:** Modeling *Nephropathia epidemica* Incidence and Bank Vole Density Over Time Through Generalized Additive Models (GAM).

Model	∆qAIC
*Generalized additive model (GAM) bank vole density over time*	
Spring	
No autocorrelation function	17.5
Bank vole density 1 year earlier	19.5
Bank vole density 1 and 2 years earlier	**–**
Bank vole density up to 3 years earlier	1.6
Autumn	
No autocorrelation function	25.4
Bank vole density 1 year earlier	24.5
Bank vole density 1 and 2 years earlier	1.7
Bank vole density up to 3 years earlier	–
*Generalized additive model (GAM) Nephropathia epidemica incidence over time*	
Summer	
No autocorrelation function	11.4
Incidence 1 year earlier	12.1
Incidence 1 and 2 years earlier	**–**
Incidence up to 3 years earlier	1.6
Winter	
No autocorrelation function	7.2
Incidence 1 year earlier	11.5
Incidence 1 and 2 years earlier	**–**
Incidence up to 3 years earlier	7.9

The choice of autocorrelation function for each model and season was based on qAIC comparisons (c.f. methods in the manuscript). We chose the model with the lowest qAIC value. If two or more models differed by less than two qAIC units, we chose the simplest mode.

Overall PUUV prevalence in bank voles in spring 1980–1986 was 27.5% (mean annual prevalence = 27%, standard error (SE) = 2.8) and was 47% in spring between 2004 and 2013 (mean annual prevalence = 34%, SE = 6.8) (Fig. 1b). In autumn, overall prevalence was 19% in 1979–1986 (mean annual prevalence = 17%, SE = 2.6) compared to 16.5% in 2003–2013 (mean annual prevalence = 17%, SE = 2.4). Also, variance in prevalence in spring in 2004–2013 was higher than variance in 1980–1986, but not in autumn (*F*_6,9_ ratio= 0.12, *p* < 0.05 for spring, *F*_6,10_ ratio = 1.32, *p* = 0.66 for autumn).

Spring PUUV prevalence in bank voles was dependent on bank vole density during current spring and previous autumn (pseudo *R*^2^ =83%*, p *< 0.01 for both predictors) in the five cycles between 1980 and 1986 (III and IV) and 2004–2013 (X, XI, XII). Autumn prevalence, however, was not significantly related to current or previous bank vole density (*p *= 0.93).

**Figure 2:**
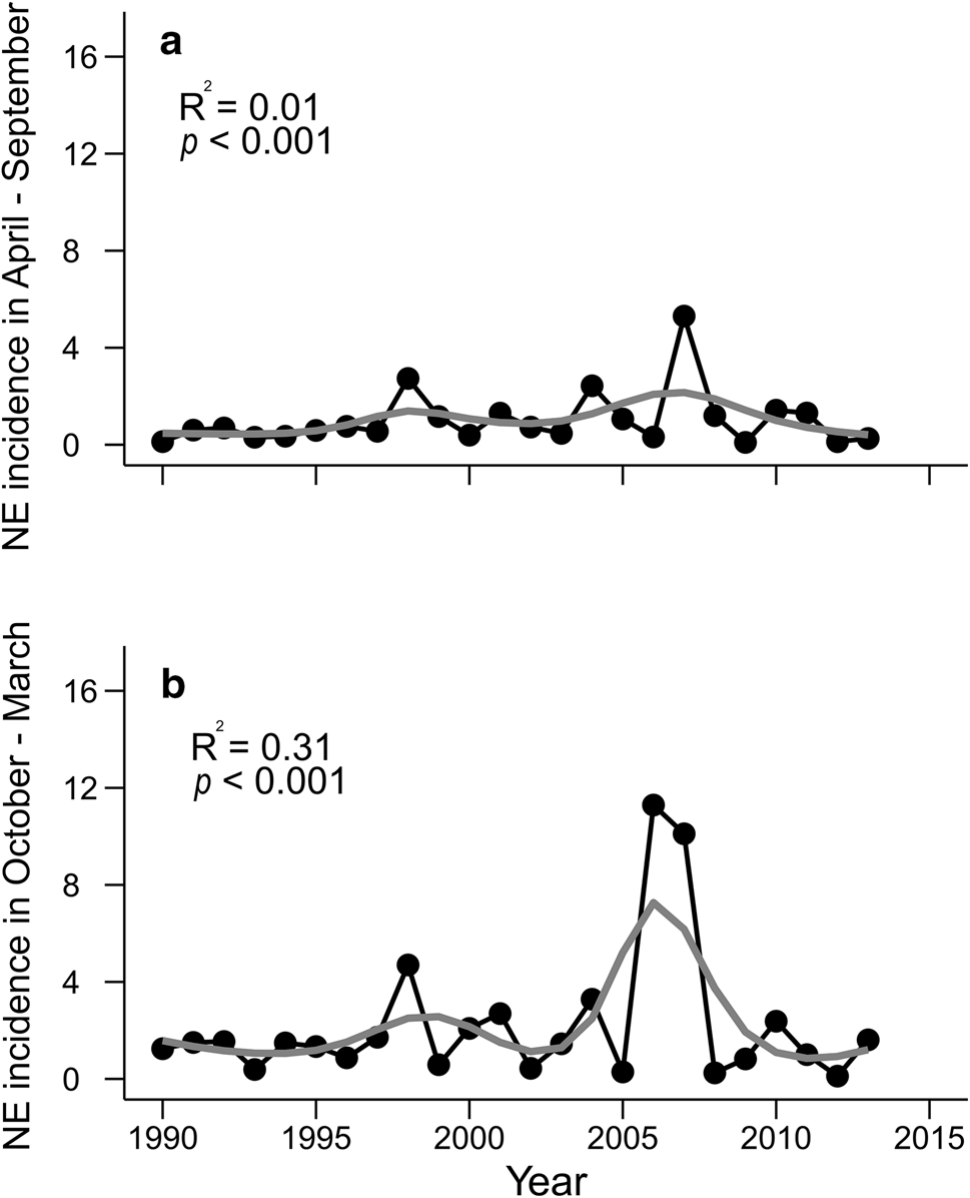
*Nephropathia epidemica* (NE) incidence in northern Sweden (no. cases/100,000 inhabitants) in **a** spring–summer (April–September) and **b** autumn–winter (October–March) 1990–2013. The gray line represents the fitted GAM model.

Between 1990 and 2013, NE incidence generally mirrored the changes in bank vole density (Fig. 2, GAM: *F*_20.4_= 16.79; *p* < 0.001 for summer, *F*_21.5_= 24.96; *p* < 0.001 for winter). The best model for summer and winter incidence included a temporal autocorrelation term with a two-year lag (SI Table 2). The temporal autocorrelation in NE incidence was likely due to its dependence on cyclic vole densities, and thus NE incidence displayed a cyclic pattern itself.

**Figure 3:**
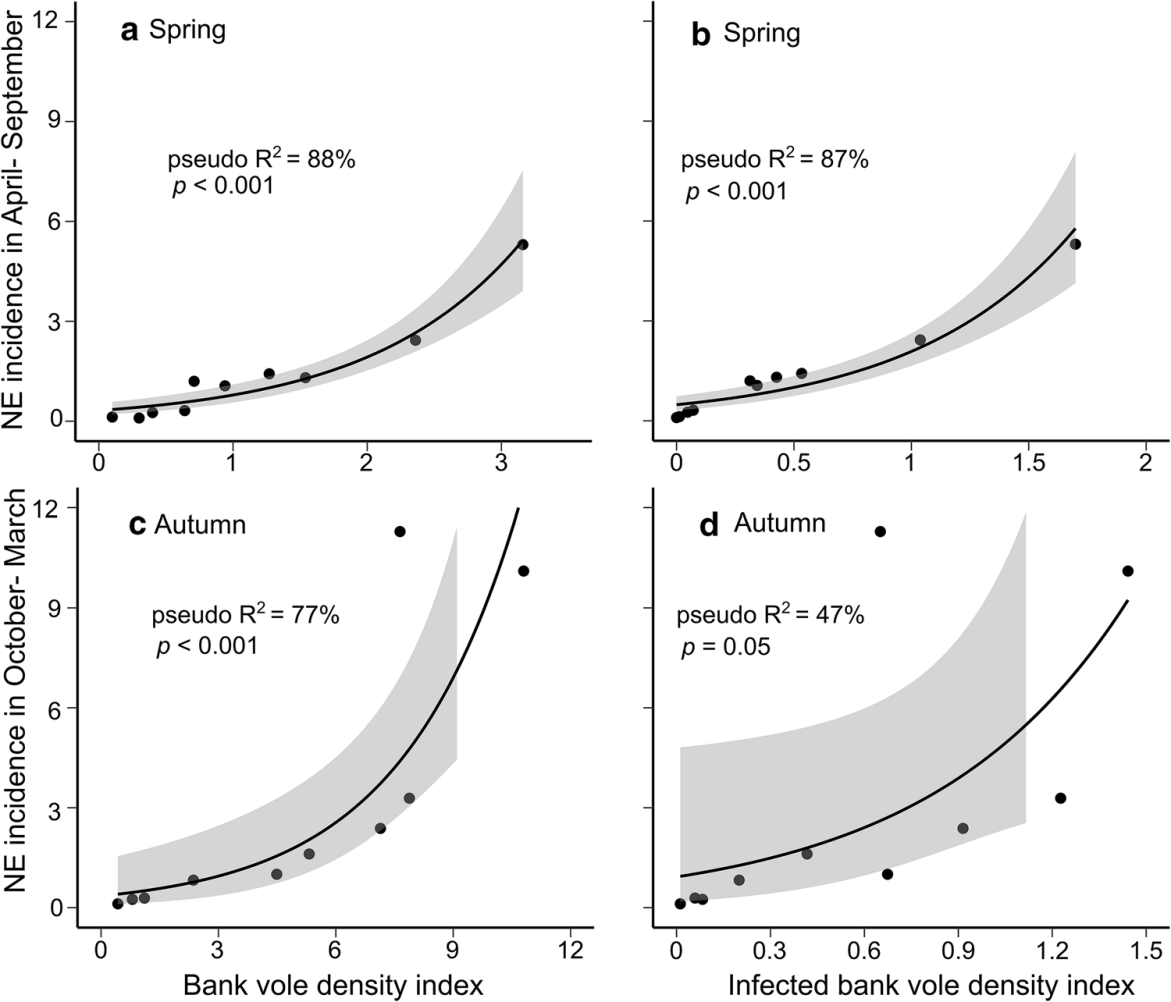
Explaining *Nephropathia epidemica* (NE) incidence in northern Sweden (no. cases/100,000 inhabitants), in **a**, **b** April–September and **c**, **d** October–March using overall density of bank voles (**a**, **c**) and density of infected bank voles (**b**, **d**) in spring and autumn. The black lines correspond to fitted generalized linear models with quasi-Poisson error distribution, and the gray shaded area represents standard error around fitted line.

Overall bank vole density and density of infected voles in spring predicted NE incidence well in summer (Fig. 3a, b), explaining 88% (Odds Ratio (OR) = 2.42; CI = 1.95–3.01; *df* = 8; *p* < 0.001) and 87% (OR = 3.68; CI = 2.66–5.11; *df* = 8; *p *< 0.001) of the variation (pseudo-*R*^2^), respectively, and ∆qAIC was < 2. Likewise, in winter, bank vole density in autumn significantly predicted NE incidence and explained 77% (OR = 1.38; CI = 1.20–1.60; *df* = 9, *p* < 0.01, Fig. 3c) of the variation, but density of infected bank voles was borderline nonsignificant with ∆qAIC > 10 and only explained 47% (OR = 4.23; CI = 1.12–16.00; *df* = 9, *p *= 0.06) of the variation in NE winter incidence (Fig. 3d). Interestingly, the increase in NE incidence with bank vole density appeared almost linear in summer but exponential in winter (Fig. 3a, c).

**Figure 4:**
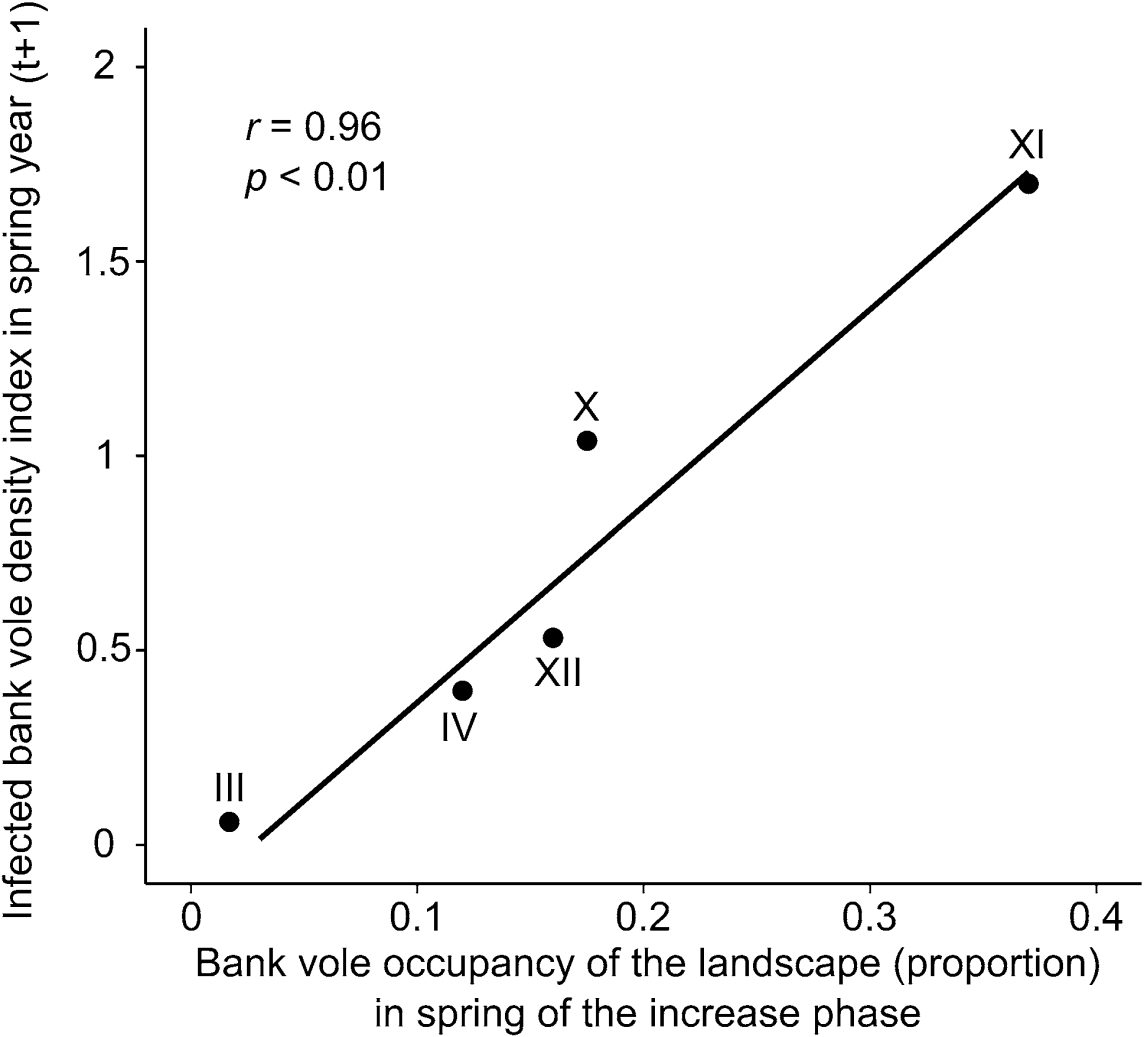
Dependence of infected bank vole density in spring year *t *+ 1, in the five cycles with infection data (III, IV, X, XI, XII), on bank vole occupancy of the landscape (proportion) in spring of the increase phase, when average autumn density had reached > 1 individual per 100 trap nights. *r* is Pearson’s correlation coefficient.

**Figure 5:**
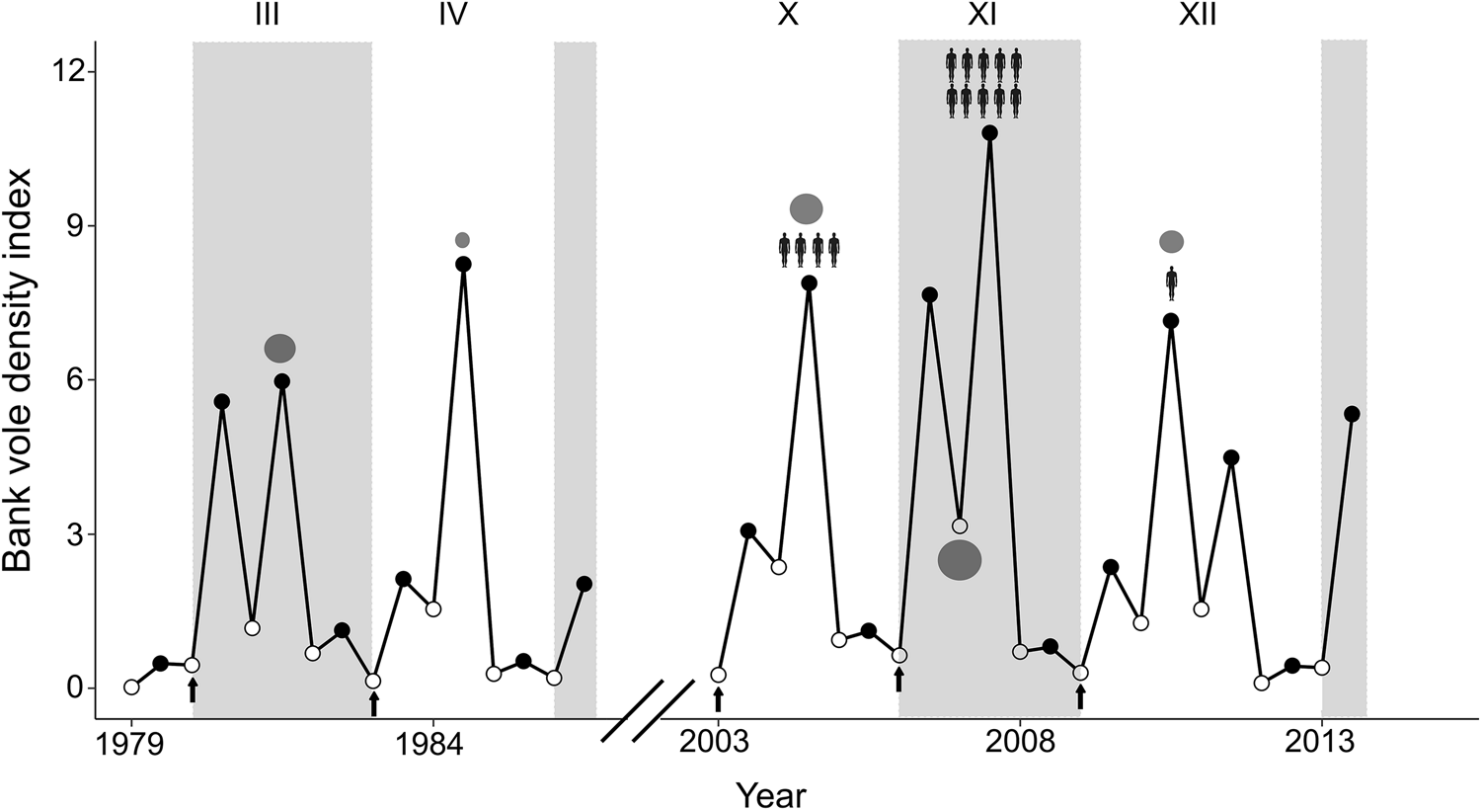
Bank vole density (number of trapped individuals per 100 trap nights) in different cycles (every other cycle is shaded) in 1979–1986 and 2003–2013 in spring (open circles) and autumn (filled circles). The size of the gray circles is proportional to the maximum density of infected voles and positioned to indicate when that density was reached in a cycle (133, 72, 113, 161, 88, infected bank voles in 1981, 1984, 2004, 2007, 2010, respectively). Arrows indicate springs when we forecast the maximum density of infected bank voles, typically 18 months earlier. Human infection data were available for 2003-2013 (*n* = 3 cycles; see methods), and the number of human silhouettes is proportional to annual incidence of *Nephropathia epidemica* in northern Sweden (no. cases/100,000 inhabitants) in July to following June in the year with maximum density index of infected bank voles (466, 1278, 128 cases in 2004, 2007, 2010, respectively).

Bank vole density in the spring of the increase phase could predict the maximum attained density of infected voles—typically 18 months later—during the same cycle (*t* = 5.4, *df* = 3, *p *= 0.01). The predictive *R*^*2*^, calculated from PRESS, was 80%. However, this result needs to be interpreted with caution, given that we only had data for five vole population cycles. Similarly, bank vole occupancy of the landscape, expressed as proportion of occupied sampling plots during spring of the increase phase of the cycle, was strongly correlated with density of infected bank voles the following spring (Fig. 4, *Pearson r* = 0.96, *t* = 4.3, *df* = 3, *p *< 0.05). However, this relationship also reflects the influence of overall bank vole density, as density and spatial distribution of bank voles in the landscape are strongly correlated (Khalil et al. 2014b). The highest NE incidence in each vole population cycle reflected initial overall bank vole density and maximum attained density of infected animals (Fig. 5).

## Discussion

Overall bank vole density and the density of infected bank voles in spring predicted NE incidence in summer accurately, whereas overall bank vole density in autumn, but not density of infected animals, was a good nonlinear predictor of NE incidence in winter. Our results also suggest that the highest density of infected bank voles in a population cycle, contributing to peak NE risk, can be predicted at the beginning of that cycle, approx. 18 months earlier.

### Long-Term Trends in Bank Vole Density

Previous studies reported dramatic declines of field vole (*Microtus agrestis*) and gray-sided vole (*Myodes rufocanus*) populations during the past three decades (Hörnfeldt 2004; Cornulier et al. 2013; Magnusson et al. 2015a). Bank vole populations also declined, but the decline was partly reversed, and peak density of population cycles increased during the 2000s (Fig. 1a). In Central Finland, similar changes in bank vole dynamics occurred, where dampened changes in density between 1995 and 1998 reverted to multiannual high-density fluctuations after 1999 (Kallio et al. 2009). These synchronous and similar patterns in Sweden and Finland suggest a form of regional influence of winter weather on population dynamics of small mammals, e.g., of field voles (*Microtus agrestis*) (Hörnfeldt 2004) and Norwegian lemmings (*Lemmus lemmus*) (Kausrud et al. 2008). Reduced competition from declining sympatric vole species and relaxed predation from declining predators may have benefitted bank vole populations (Magnusson et al. 2015a; Khalil et al. 2016), possibly facilitating the recent recovery of bank vole population.

### Bank Vole Density and Infection Prevalence

A positive correlation between prevalence and host density was reported in the PUUV—host system (Olsson et al. 2003a; Voutilainen et al. 2012)—and in other rodent-borne zoonoses, such as in plague (Davis et al. 2004). Here, we found that spring PUUV prevalence was dependent on autumn, i.e., “initial,” vole density and vole survival through the course of winter, reflected by vole density in spring. Recently, Voutilainen et al. (2016) reported that most PUUV infections in bank voles occur during winter, which is consistent with our findings here and suggests accelerated transmission. During winter, bank voles would have lost the protection provided by maternal antibodies (Kallio et al. 2010). Additionally, they lose territoriality and tend to aggregate (Ylönen and Viitala 1985), which likely increases their contact rates. Virus survival in the environment may be enhanced due to lower temperatures and high moisture levels (Kallio et al. 2006). Consequently, the rate at which susceptible bank voles are exposed to PUUV particles in the environment during winter probably increases.

PUUV prevalence and overall density of bank voles in spring were higher and fluctuated with greater amplitude in 2004–2013 compared to 1980–1986 (Fig. 1). The low spring densities between 1980 and 1986 indicate that bank vole populations declined too rapidly during winter and thus failed to sustain high levels of PUUV transmission. In such a scenario, the PUUV in bank vole system would be in disequilibrium, as high autumn density after reproduction leads to a brief increase in transmission rate, only to be offset soon after by a swift decline in host density (Luis et al. 2015). In 2004–2013, higher densities in spring suggest that PUUV transmission remained high during winter, leading to elevated PUUV prevalence (Fig. 1b).

In autumn, we found no evidence for a relationship between the density of bank voles and their infection prevalence. The influx of newborn voles during the reproductive season probably masked any increase in PUUV transmission rate with density (Niklasson et al. 1995; Lehmer et al. 2012; Roche et al. 2012). We have previously found that infection probability in autumn increases with bank vole weight, a surrogate for age (Khalil et al. 2016). An increase in infection probability with host age is typical for horizontally transmitted pathogens, including hantaviruses (Kuenzi et al. 2001; Olsson et al. 2002). In autumn, 41% of trapped bank voles weighed < 17 g and were probably younger than 12 weeks of age (Kruczek 1986), compared to 1% in spring. This demographic bias toward younger individuals, resulting in a “juvenile dilution effect,” may be responsible for the idiosyncratic relationship between density and prevalence in autumn, as voles born in the same season have not yet been exposed to PUUV or are temporarily immune due to maternal antibodies.

The 58 trapping plots in our 100 × 100 km study area provide landscape-level estimates of host density and PUUV prevalence. However, the data provide less insight on short-term temporal patterns in PUUV transmission, since sampling was only twice a year, essentially for population monitoring purposes (Hörnfeldt 1978, 1994, 2004). For example, the observed decoupling of host abundance and prevalence in autumn was most likely transient and observed through a snapshot of the relationship between density and prevalence at a time of high vole population turnover. Studies of monthly or bimonthly changes in PUUV prevalence are better suited to investigate how short-term demographic and density changes influence transmission and prevalence (e.g., Kallio et al. 2009; Voutilainen et al. 2016).

### Explaining Seasonal NE Incidence in Humans

The changes in NE incidence reflected corresponding patterns in vole density and prevalence (Figs. 1, 2). The observed increase in winter NE incidence with autumn bank vole density (Fig. 3c) is compatible with an increase in winter PUUV prevalence in bank voles. At higher bank vole densities, accelerated PUUV transmission in late autumn and winter (Voutilainen et al. 2016) would likely lead to an increase in the density of infected individuals, exacerbating human risk. Bank voles share nests (Glorvigen et al. 2012), and previously immune voles lose maternal antibodies with increased age (Kallio et al. 2006), possibly explaining accelerated transmission in winter. In spring, however, bank vole populations consisted mainly of overwintered individuals, and we suspect that the rate of PUUV transmission had already stabilized, which is supported by the almost linear relationship between spring bank vole density and summer NE incidence (Fig. 3a). Had PUUV transmission rates and recruitment of infected animals remained high during spring, we would expect a nonlinear increase in NE incidence. The relationship between spring bank vole density and summer NE incidence would then be similar to the observed relationship between autumn bank vole density and NE incidence in winter (Fig. 3c).

Ultimately, given the relationship between bank vole density and prevalence in spring, both overall density of bank voles and that of infected bank voles were satisfactory predictors of NE risk in summer. Thus, density of bank voles in spring can be used to predict human incidence in summer without any knowledge on spring infection rates, a result reported earlier (Kallio et al. 2009). In winter, bank vole density in autumn was a good nonlinear predictor of human risk, in contrast to density of infected voles, which we expect to increase rapidly as PUUV transmission accelerates during winter.

Assessing risk of zoonotic diseases that originate in wildlife entails disentangling what is often a complex ecological system. PUUV is a directly transmitted pathogen, and its transmission within host populations and to humans does not involve a vector. The relative simplicity of this pathogen–host system compared to vector-borne zoonoses such as Lyme disease (Ostfeld et al. 2006) makes it a good model system to link host density and infection dynamics to human risk. Nevertheless, factors other than bank vole density and infection rates play a role in NE epidemiology. For example, seasonal differences in human behavior and vole infestation of human dwellings are important (Olsson et al. 2003b). Winter weather, especially rain-on-snow phenomenon (Khalil et al. 2014b), may trigger bank vole movement into human dwellings to seek shelter from cold. Future studies ought to test these hypotheses by linking vole infestation and human exposure to PUUV to environmental variables such as habitat and temperature.

### Early Forecast of NE Risk

The density and landscape distribution of bank voles at low density (increase phase) at the start of population cycles can contribute to the early warning of NE risk. Bank vole landscape distribution during the increase phase of a population cycle in spring was correlated with the density of infected animals 1 year later, in spring of the peak phase (Fig. 4). While density of bank voles during the increase phase predicts the maximum density of infected bank voles reached anytime during that cycle. We only had five bank vole population cycles to base these predictions upon, which may have contributed to the high predictive performance of the model. Ongoing monitoring of bank vole populations will enable further validation of its predictive capacity.

When host density drops below a certain threshold, the pathogen may go locally extinct and infection rates take longer to build from that low level (Luis et al. 2015). In cyclic populations with predictable changes in host population trends, density minima determine the starting point of pathogen proliferation. Relatively higher host densities during those minima can act as a springboard for pathogen transmission and culminate in higher risk in the near future, when high reproductive output leads to peak density of infected animals. Our result indicates that the highest density of infected voles then contributes to maximum NE incidence during a given cycle (Fig. 5).

## Conclusions

Elucidating the relationship between host abundance and its infection prevalence on the one hand and disease incidence on the other can guide data collection for risk assessment, e.g., when to sample from host populations and what parameters are most suitable to obtain for risk prediction. In spring, when overall bank vole density and infection prevalence were positively related, bank vole density can be used to predict human risk in summer. In autumn, bank vole density and infection prevalence were decoupled, and human risk in winter was nonlinearly related to bank vole density, suggesting an accelerated transmission among bank voles in winter. To forecast the potential of an NE outbreak during a given vole cycle, bank vole density at the beginning of that cycle, i.e., 18 months earlier, may be used.
